# Castleman Disease Mimicking Pancreatic Neuroendocrine Tumour: Interpretation of Positron Emission Tomography in Pancreatic Incidentaloma

**DOI:** 10.7759/cureus.29899

**Published:** 2022-10-04

**Authors:** David T Khalil, Kellee Slater

**Affiliations:** 1 Medicine, Queensland Health, Brisbane, AUS; 2 Surgery, University of Queensland, Brisbane, AUS

**Keywords:** pancreatic neuroendocrine tumours, fluorodeoxyglucose positron emission tomography, differential diagnoses, pancreatic malignancy, castleman disease

## Abstract

We report a rare case of an incidental pancreatic lesion that proved to be Castleman disease in a peripancreatic lymph node, which mimicked a high-grade pancreatic neuroendocrine tumour (PNET) based on findings on positron emission tomography (PET). The disease was discovered as an incidental finding on CT imaging of the abdomen and was investigated and managed as PNET. Surgical resection was performed with distal pancreatectomy and splenectomy, however, histology revealed the lesion was a lymph node affected by Castleman disease. Often termed the great mimic, Castleman disease is a rare lymphoproliferative disorder that is often mistaken for other primary lesions of the organ due to its location and should be considered a differential of fluorodeoxyglucose (FDG)-avid PET lesions on imaging.

## Introduction

Pancreatic lesions discovered incidentally after imaging are a frequent event and are occurring more often as the number of scans being performed is increasing. Accurate pre-operative diagnosis of these lesions is challenging due to the position of the pancreas in relation to the gastrointestinal and vascular structures surrounding it.

Preoperative lesion characterisation is becoming essential, as our understanding of the biology of pancreatic incidentalomas increases. Lesions with a more benign-behaving trajectory can be managed by active surveillance, whereas more aggressive lesions should be managed by surgery.

Of the malignant pancreatic neoplasms, 85% of lesions are of exocrine origin, the most common being adenocarcinoma [1]. Because these tumours usually present late, the five-year survival rate is approximately 6% [2,3]. These tumours are usually straightforward to diagnose on computed tomography (CT) scans and biopsy with endoscopic ultrasound (EUS). Less commonly, pancreatic malignancies are pancreatic neuroendocrine tumours (PNET). These tumours demonstrate a spectrum of malignant behaviour. Grade I-II tumours have a five-year survival rate of 61.9%, with the potential to be observed, while Grade IV tumours have a propensity to spread and a five-year survival rate of less than 20%. These high-grade tumours generally require surgery and chemotherapy [4].

Nuclear medicine scans have become critical in differentiating and staging PNETs [5]. PNET tumours will be avid using Gallium-68 DOTATATE positron emission tomography (D-PET) and PET utilising the isotope fluorodeoxyglucose (FDG-PET) will further differentiate these tumours into high grade and low grade [6].

The stakes for the pre-operative diagnosis of incidental pancreatic lesions are further raised by the morbidity and potential mortality associated with Whipple’s procedure or distal pancreatectomy to treat these lesions [5,7]. Similar to EUS, which has a relatively high false negative and non-diagnosis rate, PET is a relatively new modality that also has a failure rate and may be positive with other conditions, both malignant and benign [8,9].

We describe a case of pancreatic incidentaloma that proved to be Castleman disease involving a peripancreatic lymph node where the pre-operative diagnosis and treatment were directed by the PET scan results.

## Case presentation

**Figure 1:**
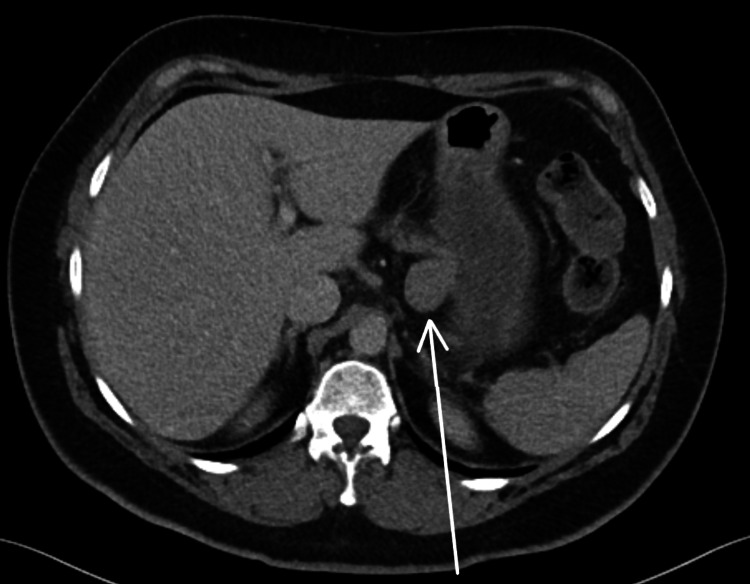
CT scan of abdomen and pelvis. Arrow pointing to the lesion of suspicion.

The patient was a 61-year-old female who presented to the emergency department with a 10-day history of vomiting, diarrhoea and abdominal discomfort. The symptoms were attributed to a viral illness but a CT scan of the abdomen was performed to exclude other pathology. This revealed an incidental 2.8 cm lesion abutting the superior border of the pancreas and adjacent to the lesser curvature of the stomach (Figure 1). It was unclear whether the lesion was arising from the pancreas or the stomach. She was referred to our hepato-biliary unit for further workup. The patient had undergone a CT scan two years earlier for other reasons. Although not reported, the lesion was present at the time and was 2 mm smaller. Magnetic resonance imaging (MRI) confirmed the lesion but provided no new information.

Because of the location of the lesion, the differential diagnosis was wide. A gastrointestinal stromal tumour (GIST) of the stomach, PNET, lymphoma, pancreatic adenocarcinoma and splenunculus were all considered. The patient was sent for an EUS that demonstrated a 21x11 mm lesion that appeared separate from the stomach and arising from the pancreas. The lesion was homogenous and hypoechoic without the classical appearance of a gastrointestinal stromal tumour. Fine-needle aspirate biopsies were suggestive of a lymph node with no other specific features. Given the patient’s gastrointestinal symptoms, serum vasoactive intestinal peptide, chromogranin A and gastrin levels were tested, all of which were within normal limits.

Given the difficulty in determining the tissue origin of the lesion, a diagnostic laparoscopy was performed. The mass was a 2 cm, well-circumscribed lesion and appeared to originate from the superior border of the pancreas. Its macroscopic appearance was that of a neuroendocrine tumour. Pre-operatively, the patient was adamant she wanted the lesion removed if it was found to be arising from the pancreas. However, the laparoscopy occurred the day Australia locked down for coronavirus disease 2019 (COVID-19). We decided, because of the slow growth over a long interval and the uncertainty of the coming weeks, to not proceed to distal pancreatectomy or enucleation and perform a PET scan to further characterise.

**Figure 2:**
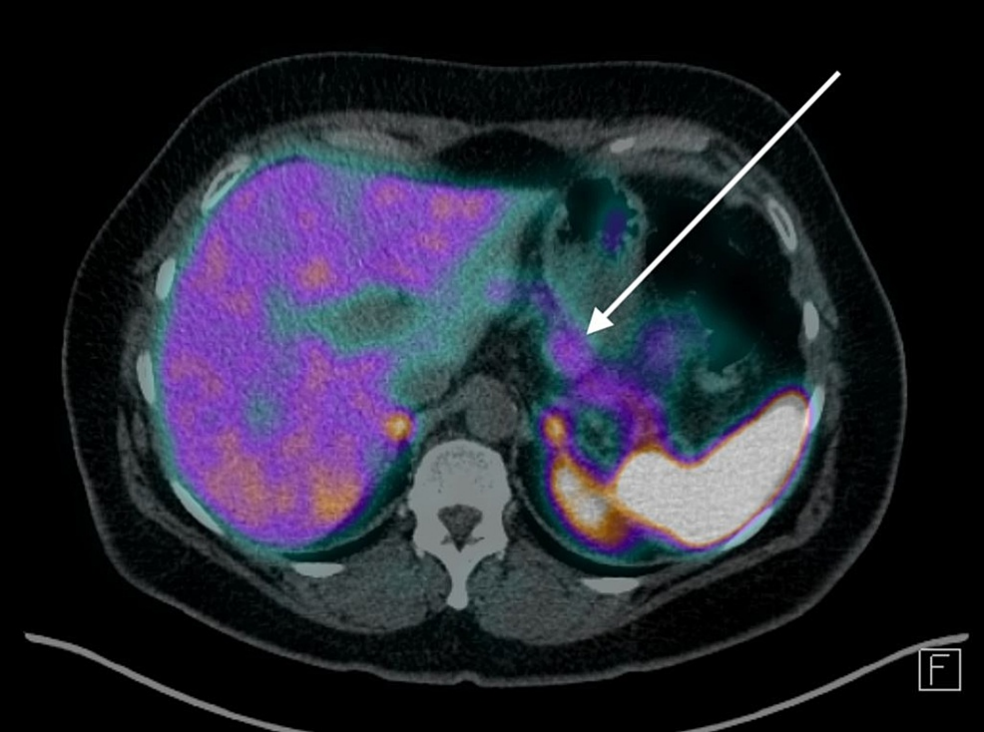
D-PET scan The white arrow indicates an avid lesion in the body of the pancreas. D-PET: DOTATATE positron emission tomography

**Figure 3:**
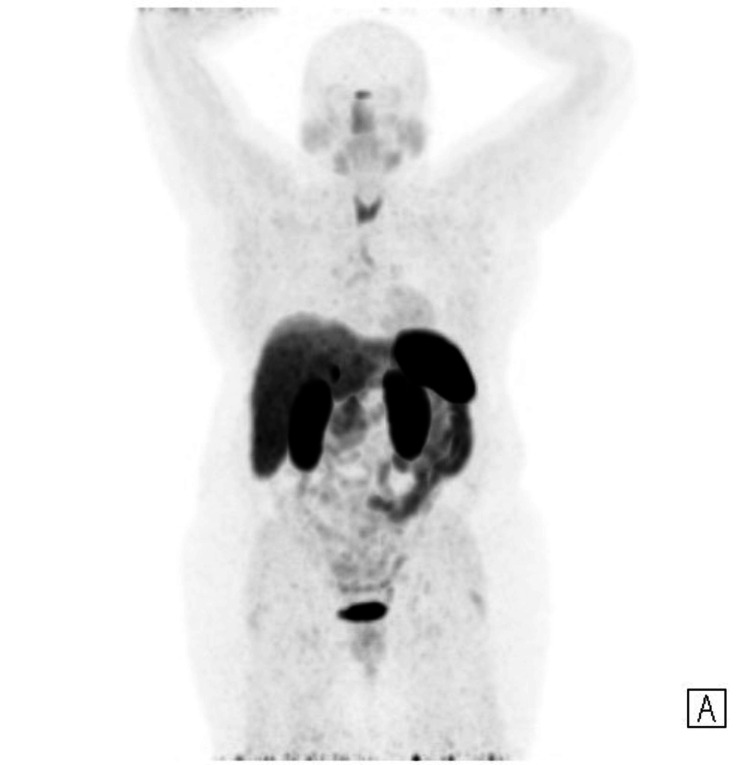
D-PET full body maximal intensity projection image (MIP) D-PET: DOTATATE positron emission tomography

**Figure 4:**
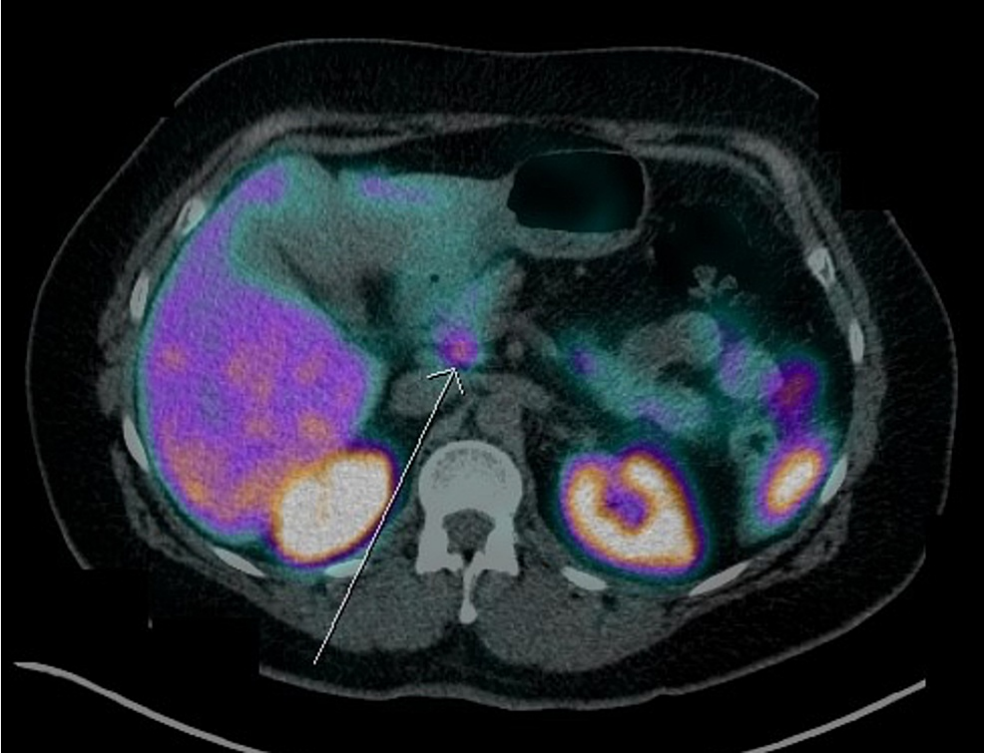
D-PET scan highlighting uptake in the head of the pancreas (arrow) D-PET: DOTATATE positron emission tomography

A D-PET scan was then performed (Figures 2, 3), confirming an exophytic lesion arising from the ventral aspect of the body of the pancreas with DOTATATE expression higher than the rest of the pancreas. A further focus of increased DOTATATE expression at the posterior head of the pancreas/uncinate was seen, which initially was thought to be due to a pathological lymph node (Figure 4).

**Figure 5:**
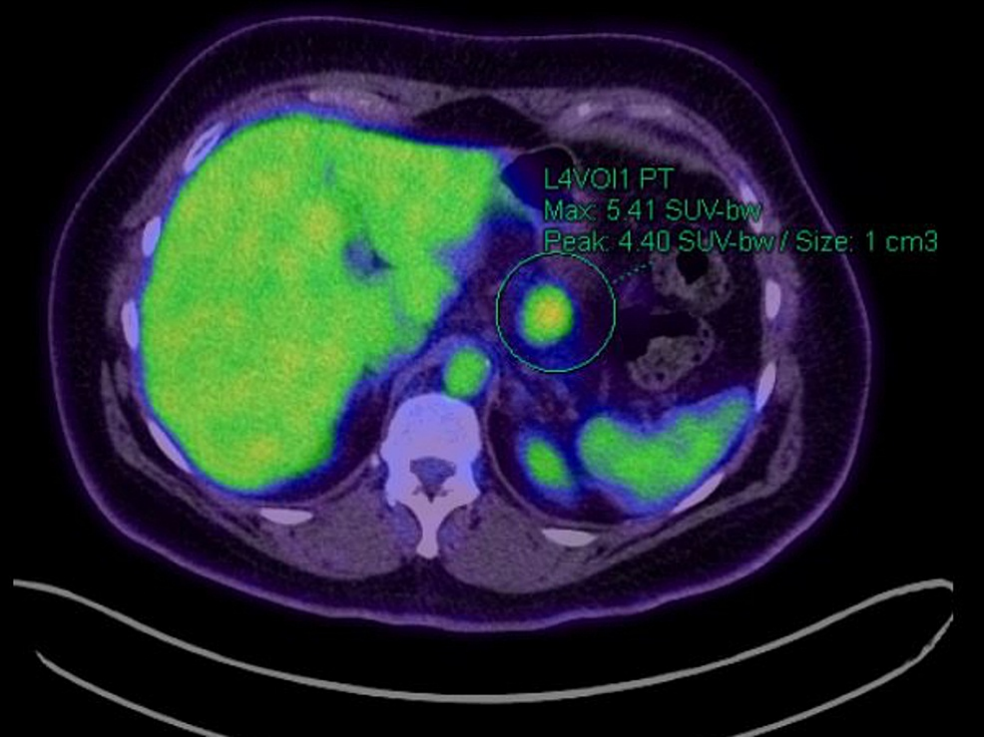
FDG-PET scan Green circle highlighting the pancreatic lesion being investigated. FDG-PET: fluorodeoxyglucose-positron emission tomography

**Figure 6:**
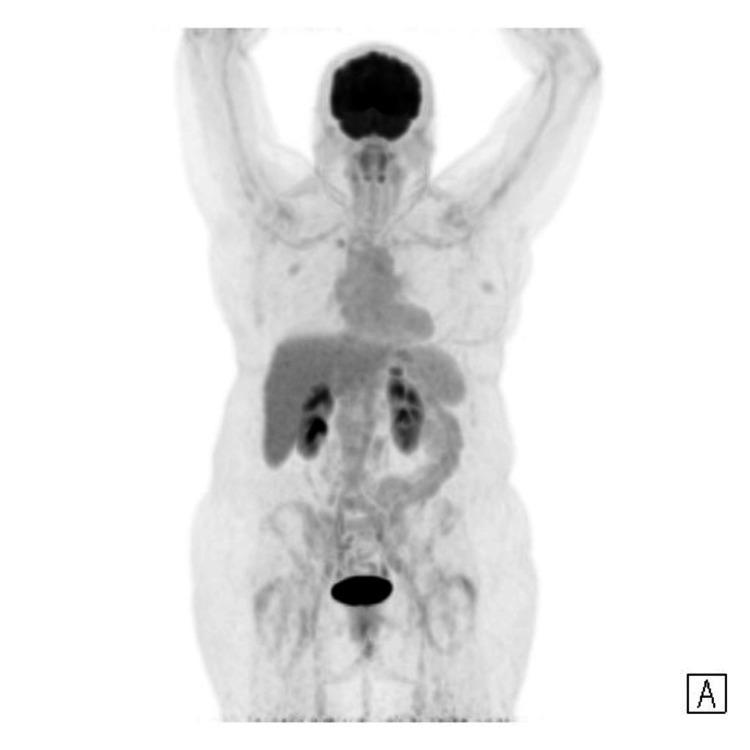
FDG-PET full body maximal intensity projection image (MIP) FDG-PET: fluorodeoxyglucose-positron emission tomography

FDG-PET was also performed (Figures 5, 6), revealing a lesion in the body of the pancreas demonstrating intense avidity, suggestive of a high-grade neuroendocrine tumour. There was no FDG uptake in the head of the pancreas, corresponding to the area of uptake on the DOTATATE scan, indicating this was a physiological uptake rather than pathology.

These findings were discussed at the multidisciplinary team meeting and the diagnosis remained inconclusive. The provisional diagnosis was FDG-avid PNET. The patient proceeded to surgical excision of the lesion via laparoscopic distal pancreatectomy and splenectomy.

**Figure 7:**
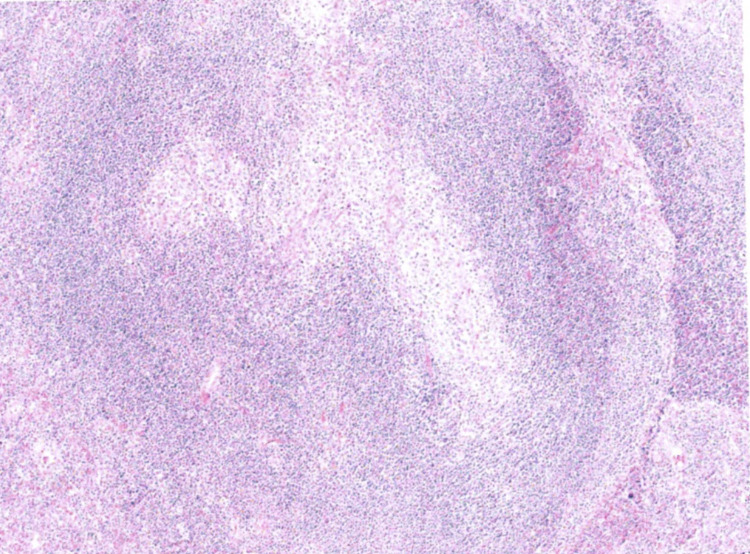
Microscopy of the lesion demonstrating the hyalinised follicles with a prominent layered mantle zone, typical of hyaline-vascular Castleman disease

The specimen was sent for histology and revealed that the lesion was Castleman disease affecting a lymph node embedded in the pancreas (Figure 7).

The patient has now had 12 months of follow-up and is recurrence-free.

## Discussion

Often termed “the great mimic”, Castleman disease (CD) was first described in the 1950s in published case reports. Benjamin Castleman, after whom the disease is named, discovered this unusual lymphoproliferative disorder characterised by foci of hyalinisation in the follicles [10,11].

CD can be classified as either unicentric or multicentric, depending on how many lymph nodes are involved. The more common unicentric CD (UCD) is a disease with an unclear aetiology, and, unlike multicentric CD (MCD), is not associated with human immunodeficiency virus (HIV) or human herpesvirus 8 (HHV-8) [12]. There are different histologic subtypes: hyaline vascular being most commonly associated with UCD and plasma cell subtype associated with MCD. MCD is more frequently associated with systemic symptoms, such as night sweats, fatigue or weight loss, but UCD is frequently asymptomatic and is usually an incidental finding on imaging [13].

Clinicians have attempted to describe the spectrum of appearance of CD on imaging. In one study, it is described as a well-circumscribed solitary lesion that enhances on contrast imaging [14]. As we found in this case, like other lymphoproliferative disorders, these lesions generally have moderate FDG uptake on a PET scan [15].

Despite imaging findings similar to that described in the literature, there was a diagnostic difficulty in this case due to a number of factors. First, given the location of the node, the differential diagnoses favoured a pancreatic lesion rather than a nodal lesion. In the literature, it has often been described that CD lesions are found embedded within the parenchyma of an organ. Hence, this disease may emulate inflammatory or neoplastic lesions related to that specific organ on imaging, as seen in this case. For this reason, CD is frequently termed “the great mimic.” Additionally, even if a nodal lesion was suspected, the provisional diagnosis of FDG-avid lymph nodes without an obvious primary is usually B-cell lymphoma or other forms of lymphoproliferative disorders. Given the rarity of CD, it is seldom considered in the differential of such presentations [16-18].

Fortunately, UCD has a high cure rate with complete resection, and surgery is the mainstay of treatment. The overall survival after 10 years with complete resections is greater than 95%, and failure to treat was associated with significant mortality [19]. Often due to size or location, complete resection is not possible and the efficacy of neo-adjuvant radiotherapy in reducing tumour size in a non-resectable disease has been described in the literature, with the aim of making complete resection possible [20].

## Conclusions

Timely and accurate workup and management of incidental pancreatic masses is critical and remains a challenge for clinicians. Unicentric Castleman disease has often been misdiagnosed as a primary lesion of the organ of its location, largely because of the rarity of the disease, and this was also evident in the above-described case of pancreatic Castleman disease. Although rare, it should be considered a differential diagnosis in PET-avid lesions in the pancreas and other primary organ masses.
